# Impaired Intestinal Barrier and Tissue Bacteria: Pathomechanisms for Metabolic Diseases

**DOI:** 10.3389/fendo.2021.616506

**Published:** 2021-03-09

**Authors:** Lucas Massier, Matthias Blüher, Peter Kovacs, Rima M. Chakaroun

**Affiliations:** ^1^ Medical Department III – Endocrinology, Nephrology, Rheumatology, University of Leipzig Medical Center, Leipzig, Germany; ^2^ Department of Medicine (H7), Karolinska Institutet, Karolinska University Hospital, Huddinge, Stockholm, Sweden; ^3^ Helmholtz Institute for Metabolic, Obesity and Vascular Research (HI-MAG) of the Helmholtz Zentrum München, University Hospital Leipzig, University of Leipzig, Leipzig, Germany

**Keywords:** obesity, metabolic disease, intestinal permeability, microbiome, zonulin, adipose tissue microbiota, type 2 diabetes, endotoxemia

## Abstract

An intact intestinal barrier, representing the interface between inner and outer environments, is an integral regulator of health. Among several factors, bacteria and their products have been evidenced to contribute to gut barrier impairment and its increased permeability. Alterations of tight junction integrity - caused by both external factors and host metabolic state - are important for gut barrier, since they can lead to increased influx of bacteria or bacterial components (endotoxin, bacterial DNA, metabolites) into the host circulation. Increased systemic levels of bacterial endotoxins and DNA have been associated with an impaired metabolic host status, manifested in obesity, insulin resistance, and associated cardiovascular complications. Bacterial components and cells are distributed to peripheral tissues *via* the blood stream, possibly contributing to metabolic diseases by increasing chronic pro-inflammatory signals at both tissue and systemic levels. This response is, along with other yet unknown mechanisms, mediated by toll like receptor (TLR) transduction and increased expression of pro-inflammatory cytokines, which in turn can further increase intestinal permeability leading to a detrimental positive feedback loop. The modulation of gut barrier function through nutritional and other interventions, including manipulation of gut microbiota, may represent a potential prevention and treatment target for metabolic diseases.

## Introduction

While the obesity and type 2 diabetes (T2D) pandemics are increasing at a fast pace (1), new factors relevant to the lack of adaptation toward the increasingly rapid changes in our environment have been proposed as possible perpetrators. Among those, the intestinal microbiota and its interactions with host metabolism and the immune system have been acknowledged to influence and contribute to several diseases including gastrointestinal disorders such as inflammatory bowel diseases (2) and more recently the plethora of metabolic and cardiovascular diseases (3, 4). However, the underlying mechanisms are still unknown (5–7). One poorly understood feature of metabolic disease is the alteration and dysfunction of the intestinal barrier, accompanied with an increase in intestinal permeability. The healthy gut barrier constitutes a crucial boundary protecting the host from external stimuli and pathogens by providing spatial compartmentalization and various defense mechanisms, while concurrently allowing the uptake of necessary nutrients (8). While the influx of microbial products has been suggested to underlie chronic inflammation observed in metabolic disease and more specifically T2D (9), clinical features of metabolic diseases such as hyperglycemia in T2D have been associated with increased influx of microbial products in humans reflective of glucotoxicity (10). First studies in the 1950s suggested that increased endotoxins, which are lipopolysaccharides of gram-negative bacteria in the circulation result from an increased intestinal permeability (11). Although still under debate, independent studies in mice and humans could confirm these results (12–16). In 1984, the term “leaky gut” was first introduced when Bjarnason et al. reported that patients with excessive alcohol consumption had increased intestinal permeability (17), which is also referred to as hyperpermeability (18). In the following years research on intestinal permeability focused on autoimmune and inflammatory diseases such as celiac disease (19, 20) or Crohn’s disease (21). Only recently, possible associations between intestinal permeability and characteristics of a metabolic disease like obesity or T2D have emerged as plausible and attractive targets in the field of obesity research.

In this review, we aim to highlight underlying mechanisms of an impaired intestinal barrier and its possible impact on metabolic health. We specifically discuss recent findings on how endotoxemia and translocation of bacteria, bacterial genetic material and products may cause tissue and organ dysfunction subsequently contributing to metabolic diseases.

## The Intestinal Barrier

### Components of Intestinal Barrier

**Figure 1:**
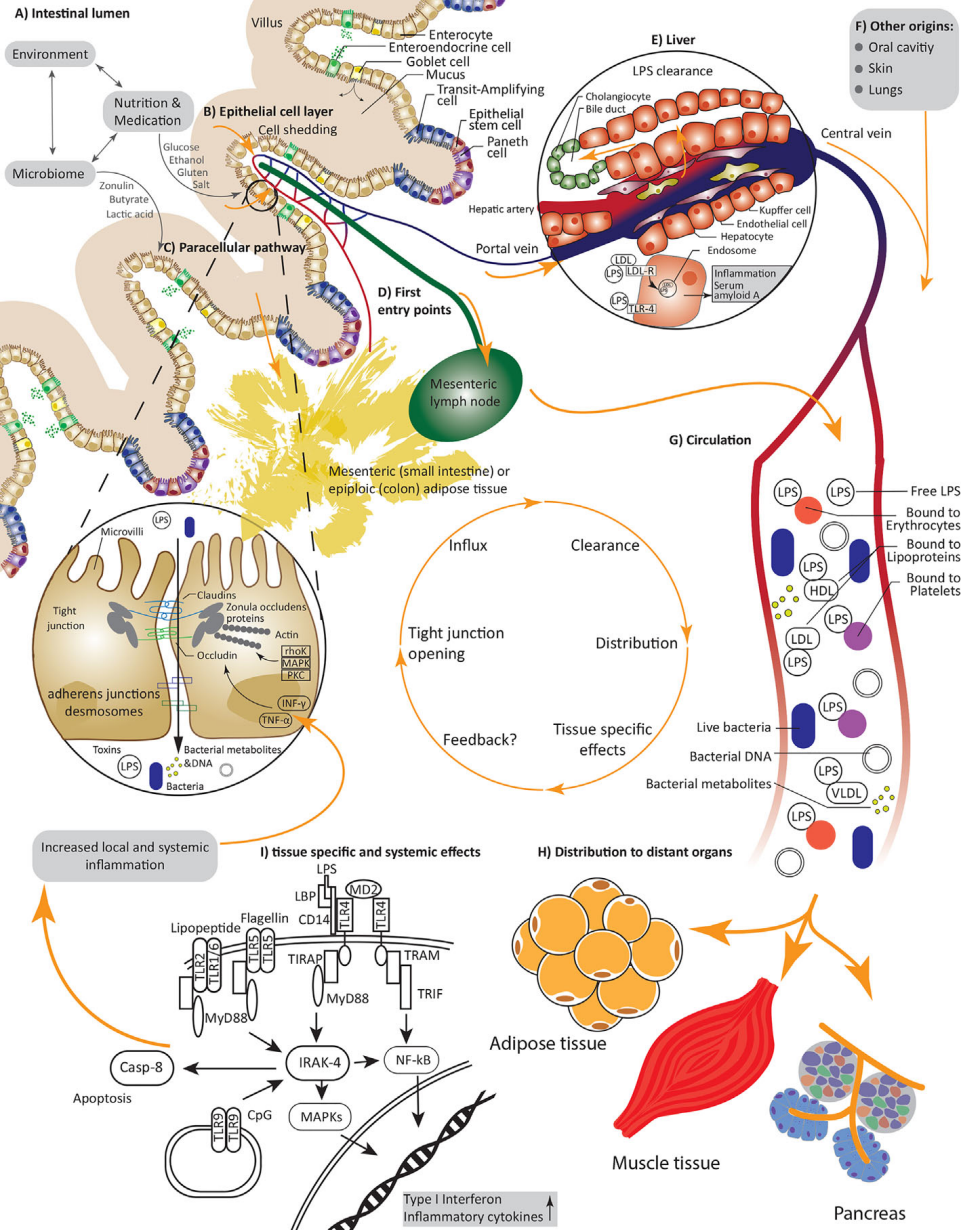
Causes and consequences of increased intestinal permeability; **(A)** An interplay between environmental factors, nutrients, drugs and the microbiome defines our intestinal lumen and can directly or indirectly alter permeability of the epithelial cell layer; **(B)** Increased cell shedding under pathophysiological conditions or the reversible interaction with and opening of tight junctions under allow an influx of pathogens including bacteria or their nucleic acids, metabolites, and lipopolysaccharides as well as further substances and toxins; **(C)** Paracellular transport *via* tight junctions is regulated by various kinases and inflammatory cytokines, nutrients and bacteria can interfere with claudin and occludin expression; **(D)** First entry points of invading substances are capillaries and lymph vessels of the villi and tissues in close proximity such as mesenteric adipose tissue; **(E)** Endotoxin is rapidly and constantly cleared in the liver; **(F)** Next to the gut lumen, pathogens could also invade through the oral cavity, the skin and the lung; **(G)** Under constant interactions and clearance with the immune system the LPS and bacteria are transported in the circulation, thereby LPS can be bound to various proteins and cells including erythrocytes and lipoproteins; **(H)** Effected distant organs include adipose tissue, pancreas tissue and possibly muscle tissue; **(I)** Pathogens interact with many receptors, i.e., LPS is recruited to TLR-4 leading to increased expression and secretion of inflammatory cytokines.

The intestine represents an active interface, where external environmental factors, such as diet, medication and the billions of symbionts inhabiting our gut co-exist and interact – usually peacefully – with host factors including the immune system (**Figure 1A**). The intestinal barrier allows and facilitates the uptake of nutrients and water from the intestinal lumen, whilst at the same time providing effective mechanisms to combat harmful substances and pathogens and prevent their translocation into the host circulation and peripheral tissues (22) (**Figure 1B**). These checkpoints comprise an intricate network of mechanical and immunological factors including the mucus, the epithelium and the underlying lamina propria (22) as well as humoral and immunological factors. The goblet cells produced mucus layer constitutes of mucin and forms a protective barrier, limiting the amount of bacteria reaching the epithelial cells (23). Epithelial cells form microvilli, protrusions in the range of 100 nm width, that help to increase the area for absorptions of ions and nutrients using specified receptors (24). This line of densely arranged microvilli is termed brush border, which, on the one hand, physically prevents bacteria coming in contact with the cell soma, but otherwise help to actively combat pathogenic bacteria (25). For example, native luminal vesicles containing intestinal alkaline phosphatase are released from the tips of the microvilli, helping to dephosphorylate lipopolysacchradies (LPS), hinder bacterial growth and prevent adherence of enteropathogenic *E. coli* to the epithelial cell layer (26). Epithelial cells are connected by tight junctions (TJ), which hinder the entry of pathogens while regulating the paracellular flow of water, small ions and nutrients (27). Tight junction complexes mainly consist of occludin and members of the claudin family. Expression of the latter is tissue-specific and the claudin composition defines the kind and function of a specific tight junction, e.g., claudin-7 reduces permeability to anions and claudin-1 reduces permeability to cations (28, 29). Claudins and occludin are connected to the cytoskeleton by scaffolding proteins including zonula occludens (ZO) proteins (30) (**Figure 1C**). Additionally, cells are connected by adherence junctions (for cell-cell signaling) (31) and desmosomes (for cell stability) (32). The mechanical gut barrier interacts constantly with and is highly impacted by local immune components consisting of soluble Immunoglobulin A (IgA), epithelial and Paneth cell produced, and secreted antimicrobial peptides such as alpha-defensins, lysozyme, and C-type lectin (33) providing additional protection to the epithelial layer and the crypts of Lieberkühn (34). Not less important are cells belonging to the innate and adaptive immunity system such as macrophages, dendritic cells (35), T-regulatory cells (36) and the highly dense lymphoid tissues inhabiting the lining of the gut but also highly active in adjunct lymph nodes (37). Disruptions in this intricately balanced system through toxins, microorganisms, nutrients, or food contaminants can lead to an alteration of the intestinal permeability, by an increased and inadequately controlled influx *via* the paracellular and transcellular pathway.

The regulation of this system can, under both physiological and pathophysiological conditions, be altered by various endogenous and exogenous factors as discussed in the following paragraph.

### Endogenous Regulation of the Intestinal Permeability

A constant challenge for upholding the integrity of the intestinal barrier is the continuous cell shedding on the tip of the villi, with a turnaround time of about five days (38). Tight junctions are redistributed to block the gap under involvement of rho kinase (39). Other signal cascades involved in tight junctions regulation under physiological conditions include protein kinase C’s (PKC) and mitogen-activated protein kinase (MAPK) pathways, which regulate protein phosphorylation and expression allowing a selective transport of molecules (40). In disease, increased proinflammatory cytokines such as interferon-γ (INF γ) and tumor necrosis factor α (TNF-α) shift the homeostatic balance (27) by downregulating the expression of claudin-1 (27) and increasing occludin and claudins-1 and -4 internalization, leading to an increasingly compromised gut permeability, as shown in T84 cells *via* immunofluorescence microscopy (41). Further mechanisms of INF-γ induced permeability in T84 cells involve phosphatiodylinositol-3-kinase/protein kinase B (PI3-K/Akt) activation and a delayed but prolonged NF-kB response (42) (**Figure 1C**). This is of particular interest, as metabolic diseases are usually attended by a chronic low grade inflammation (43), possibly initiating a detrimental positive feedback loop.

Mechanisms leading to whole bacterial translocation through the mucosal barrier remain unclear. Several studies suggest transcytosis *via* M-cells (44), which uptake large antigens and particulate matter such as viruses, bacteria, and protozoa through receptor mediated endocytosis (45). The transcellular pathway can be triggered and upheld by low concentrations of INF-γ (that are insufficient to alter the paracellular pathway), as shown by Clark et al. in Caco-2 cells (46). This uptake is also dependent on extracellular signal-regulated kinase (ERK) 1/2 and ADP-ribosylation factor (ARF)-6 signaling (25, 47).

### Exogenous Perturbation

Several external factors, including bacteria, alcohol consumption, nutrition and medication have an impact on the intestinal permeability (34). A high caloric “western” diet, in particular due to high-fat/low-fiber content (48) and food additives (49), is associated with adverse changes in the microbiome (50) and both are independently important factors in the development of metabolic diseases. Whereas pathogenic bacterial species such as enteropathogenic *E. coli* (51–53), *H. pylori* (54), *S. Typhimurium* (55), and *V. cholera* (56) increase intestinal permeability, the totality of the gut microbiota contributes equally to upholding the gut barrier health by shaping tolerogenic mechanisms controlling the responses of resident dendritic cells and macrophages (57). Pathogenic bacteria can use their type III secretion system to alter the host cytoskeleton in their favor to facilitate entry to epithelial cells (58), but also non-invasive parasites like G. lamblia can introduce adverse changes, in this case by causing microvilli shortening and TJ disruption (25, 59). Moreover, beneficial effects were reported for gut bacteria produced short chain fatty acids (60, 61), in particular butyrate (61, 62), which has been shown to act as an energy source for colonocytes (63), regulate hypoxia induced factor 1α (HIF-1α) dependent expression of many pro-barrier genes in intestinal epithelial cells (64), facilitate tight junctions assembly (61) and induce colonic regulatory T-cells, leading to the suppression of inflammatory and allergic responses (62). Taxonomic groups-wise, *A. muciniphila* (65), Bifidobacterium (66), and lactic acid producing bacteria like *L. plantarum* (67) and *L. reuteri* (68) were, among others, also reported to improve intestinal permeability (**Figure 1A**).

Experimental evidence for the effect of food components mostly stems from rodent and cell culture experiments. Glucose, well known for its many adverse effects in metabolic disease (69), is partially transported paracellularly and enhances small intestinal permeability (70), increases Caco-2 cell permeability and leads to altered TJ arrangements (71). Recently, Thaiss et al. demonstrated a key role for hyperglycemia in impairing the barrier function by increasing retrograde uptake of glucose into epithelial cells *via* the glucose transporter (GLUT)-2 transporter, leading to changes in intracellular glucose metabolism and to transcriptional reprogramming. The latter includes, among others, alterations in the expression of genes involved in N-glycan biosynthesis and pentose-glucuronate interconversion, two pathways linked to the maintenance of epithelial barrier function (10). Further food components increasing TJ permeability include salt (72) and various fatty acids (73), all of which are known to contribute to obesity development (74, 75). Another important nutritional factor affecting the intestinal permeability is gliadin, a component of gluten found in wheat. In mouse experiments, Lammers et al. found that gliadin increased permeability by binding to the chemokine receptor 3 (Cxcr3) (76) (**Figure 1A**). Beneficial effects on intestinal permeability were reported for casein (77) and other cheese peptides (78), vitamin D (79) as well as polyphenols (80, 81), which is in line with findings that polyphenol rich diets ameliorate metabolic disease (82). Furthermore, addition of apple-derived pectin to a high fat diet (HFD) counteracted some negative effects of HFD by preventing microbial shifts and improving barrier function due to increased expression of claudin-1 and abating metabolic endotoxemia (83).

Moreover, occludin and claudin expression are subject to circadian control, associated with regulation of intestinal permeability and susceptibility to colitis (84). This is further substantiated by the increase in intestinal permeability after circadian disruption in mice leading to promotion of alcohol induced liver disease and inflammation (85). Similarly, gut bacterial signatures related to diurnal oscillation also enable the risk stratification and prediction of T2D within 5 years of sampling (86) linking circadian control with gut microbiota profiles and intestinal permeability regulation and downstream metabolic sequalae.

## Intestinal Permeability in Metabolic Disease

### Animal Studies

An increased intestinal permeability is the key factor for the migration of toxins and bacterial components to the circulation. First evidence for a link between obesity and increased permeability was found in mouse studies. Brun et al. used Ussing chambers – a physiological system to measure the transport of ions, nutrients, and drugs across epithelial tissues including the gut (87, 88) to analyze the barrier integrity in leptin-deficient (*ob/ob*) and hyperleptinemic, but leptin-receptor mutant (*db/db*) mice, a widely used animal models in obesity-induced T2D (89). They observed a significantly increased permeability, which was more prominent in *db/db* mice. This was accompanied by an increase in portal endotoxemia and systemic inflammation parameters (90). Later, changes in the microbiome were found to be an important factor linking intestinal permeability to inflammation (91, 92). In a key study, Cani et al. could further show that a HFD resulted in increased levels of plasma endotoxin as well as a reduced expression of TJ genes ZO-1 and occludin. This effect was reduced when mice on HFD were treated with antibiotics and plasma endotoxin levels were positively correlated with markers of inflammation (Plasminogen-Aktivator-Inhibitor 1, *PAI-1*) and oxidative stress (six transmembrane protein of prostate 2, *STAMP2*) (9). It was further shown that the shift in the intestinal permeability of mice on HFD could be attributed to claudin switching, which leads to tight junction restructuring. In this case, the expression of claudin-1,-3,-4,-7, and -15 decreased, whereas expression of claudin-2 increased (93). However, as shown in *db/db* diabetic mice with induced *C. rodentium* infection, physiological countermeasures are in place to overcome initial intestinal barrier impairment. In particular, exogenous IL-22 restored mucosal host defense as indicated by histological evaluation (94).

### Human Studies

Although some studies have linked intestinal permeability with metabolic disease in humans, they should be seen with caution, as they did not employ reproducible and robust methods to test for gut permeability such as lactulose/mannitol (La/Ma) or similar tests and Ussing chambers. In 2011 Gummesson et al. reported an association between intestinal permeability and increased visceral obesity in 67 otherwise healthy women (95) and in 2012, Teixeira et al. corroborated these finding by showing that increased permeability is associated with increased BMI and insulin resistance in 40 patients with obesity and T2D (96). Damms-Machado et al. similarly reported a correlation between intestinal permeability and Homeostasis Model Assessment for insulin resistance (HOMA-IR) in 27 individuals with obesity of which 18 suffered from the metabolic syndrome (97). Furthermore, Luther et al. reported in a meta-analysis showing that nonalcoholic fatty liver disease (NAFLD) was associated with increased intestinal permeability (odds ratio increased intestinal permeability=5.1), which was even more prominent in nonalcoholic steatohepatitis (NASH) (odds ratio=7.2) (98). Challenging this hypothesis with interventions has seldom been done: A low-caloric diet of 800 kcal/day was able to significantly reduce the intestinal permeability, indicating the relevance of nutrition as a fast acting player influencing intestinal permeability (99). Further interventions leading to mechanistic insights in the link between metabolism and intestinal permeability are highly warranted.

## Metabolic Endotoxemia and the Translocation of Bacterial Genetic Material and Live Bacteria in Human Tissues: A Compartment Centered Approach

The evidence related to translocation of bacterial components, live bacteria, and bacterial metabolites into the circulation and beyond is highly dependent on the tissue studied leading to diverging degrees of confidence in the relationship between gut permeability, organ dysfunction and overall modulation of host metabolism. On their way from the gut, bacteria are enriched in mesenteric lymph nodes (MLN) (**Figure 1D**) both in health and in disease as evidenced in translocation of enteric microorganisms to MLN in patients with advanced cirrhosis (prevalence 30.8%) and comparable prevalence of bacterial translocation (~8%) in patients and healthy controls after selective intestinal decontamination (100). Consequently, it is postulated that immune cells interacting with bacteria are activated in the MLN after which they are redistributed in the blood (**Figure 1H**) (101). Similarly, bacterial products have also been implicated as mediators between the gut microbiota and peripheral organs: A major part of consumed dietary fibers are fermented by bacteria in the colon, which results in various metabolites, including short chain fatty acids (SCFA) (102). SCFA are readily absorbed by the colonocytes and only a small part is secreted (103). Production of SCFA increases with a fiber-rich diet (102), and increased circulating levels of SCFA are associated with improvements in insulin sensitivity and decreased levels of lipolysis, triacylglycerols, and free fatty acids in humans (104).

Beyond the blood, there seem to be unexpected and promising niches of bacterial translocation and bacterial targeted action are currently drawing increasing multidisciplinary attention leading to a paradigm shift in the study and definition of metabolic diseases as “non-communicable” diseases.

### Impact of Blood Borne Bacteria and Bacterial Components on Metabolic Disease

#### Lipopolysaccharides in Health and Disease

The measurement of bacterial components in the circulation allows an indirect verification of increased intestinal permeability. This link has emanated from the observation that sepsis is associated with an acute but reversible state of insulin resistance (105). To this end, most studies have focused on the measurement of LPS, i.e., endotoxins, which are molecules on the outer membrane of gram-negative bacteria. The condition of increased exposure to bacterial LPS in the blood in obesity or metabolic disease is referred to as “metabolic endotoxemia”. Fat intake is associated with an increase in postprandial LPS levels (106), which have been shown to be distributed from the gut into the circulation *via* the mesenteric lymph nodes using freshly formed chylomicrons (**Figure 1D**) (107). LPS is recognized and bound by both LPS-binding protein (LBP) and soluble cluster of differentiation (sCD14), which can also be used as marker proteins, and recruited to the TLR 4 (108). The highest proportion of LPS in the circulation is bound to lipoproteins, whereby most of LPS is bound to high density lipoprotein (HDL) (109). Smaller fractions of LPS are also bound to platelets (110, 111), monocytes (112) and erythrocytes (113, 114) or are present as free LPS (115) (**Figure 1G**). However, these observations mostly stem from models of sepsis. Endotoxin and other bacterial components taken up from the gut due to increased intestinal permeability will presumably enter the portal vein and, as a first step be transported to the liver, where they are rapidly cleared. Clearance mostly takes place in the liver after uptake *via* – among other routes – various lipoprotein receptors, e.g., LDL-R (115) and is mainly accomplished by hepatocytes and Kupffer cells to be then excreted to the bile (**Figure 1E**). Moreover, since binding of LPS with lipoproteins and chylomicrons has been shown to prevent endotoxin induced monocytes activation and secretion of proinflammatory cytokines (116, 117), the redistribution and increased content of phospholipids among the different lipoproteins has been suggested as a potential mechanism for the attenuation of the immunostimulatory effects of LPS.

LPS is detectable in low concentrations even in healthy subjects, and a single meal with high fat content already increases LPS levels (118, 119). In the last years, many studies in large cohorts reported increased levels of LPS and LBP in subjects with metabolic syndrome or T2D (120–123). Pussinen et al. analyzed LPS levels of patients with prevalent (n=537) and incident T2D (n=462) and compared them to a control group (n=6,170), of which about 20% had the metabolic syndrome. Endotoxin was significantly increased in individuals with T2D and endotoxin activity was associated with an increased risk for incident T2D, independently of other metabolic risk factors (121). Furthermore, serum endotoxin was linearly associated with the number of metabolic syndrome traits (121). Direct administration of LPS induced insulin resistance and systemic inflammation (124) and an 8-weeks long overfeeding intervention was associated with increased endotoxemia, linking overnutrition with endotoxemia and insulin resistance (125). A more recent study by Cox et al. used LPS, LBP as well as intestinal fatty acid binding protein (iFABP) to calculate a permeability risk score, which was increased in individuals with type 2 diabetes (123). In secondary complications, such as NAFLD, even higher endotoxin levels are reported (126). Mechanistic insights in the cascades underlying the effects of LPS on gut permeability and subsequent metabolic impact stem from mice studies, where subcutaneous LPS infusion has been shown to lead to an obese phenotype, comparable to that of mice on high-fat diet, including increased glucose and insulin levels as well as whole body and adipose tissue weight gain (106). Moreover, LPS application *in vitro* has been shown to result in an increase in gut permeability through tight junction dysfunction *via* a TLR4-dependent process (127), which is the most specific LPS receptor (128, 129) (**Figure 1I**).

As for the impacts of metabolic intervention on LPS: Roux-en-Y gastric bypass (RYGB) surgery has been shown to reduce LPS levels by 20 ± 5% after a 180 days follow-up period in patients with obesity and T2D (122), which could also be confirmed for sleeve gastrectomy and duodenal switch procedures (130, 131).

Most of the studies looking at LPS however neglect the fact, that LPS from different bacteria has a high heterogeneity in its chemical structure, mostly due to variation in the O-antigen polysaccharide (132, 133), and consequently, biological function (134). The latter was nicely demonstrated by Vatanen et al., showing that LPS derived from *B. dorei* counteracts the immunostimulatory activity of *E. coli* LPS (134). To our knowledge, there are no studies looking at LPS variation in metabolic disease published so far.

That being said, a reduction of intestinal permeability has been evidenced both in rat and human models of bariatric surgery (135). In rats undergoing duodenojejunal bypass surgery (DJB), intestinal permeability as assessed by Ussing chamber studies and dextran-FITC through mucosa-to-serosa flux was reduced in the alimentary and common limb as well as the colon, consistently supported by increased mucosal expression of occludin in both the alimentary and common limbs after DJB. LPS and LBP levels were not different between DJB and sham operated rats, possibly due to increased bacterial counts and compensatory increased intestinal surface in the remaining intestine. This is in contrast to data from humans, where mucosal surface was significantly reduced after surgery and occludin as well as zonula occludens expression was reduced. Congruently though, intestinal permeability as assessed by Ussing chambers was decreased owing to an increased expression of claudin-3-expression (136), further tying up reduction of microbiota-derived inflammatory mediators and rearrangement of gut barrier regulators after bariatric surgery (135).

#### Bacteria and Bacterial Fragments

In addition to LPS measurement, other bacterial components such as bacteria itself or bacterial DNA have been suggested as possible contributors to metabolic disease. The amplification of bacterial DNA allows quantification of bacterial load while subsequent sequencing allows the assessment of microbial composition and identification of specific bacterial perpetrators. There is increasing evidence for the presence of bacteria in blood, even in healthy subjects, as extensively discussed by Castillo et al. (100). Briefly, there are multiple studies reporting concurrent bacterial phyla in blood with Proteobacteria and Firmicutes being the most dominant phyla (100, 137). The first study to relate bacterial presence in the blood with metabolic disease was published by Amar et al. in 2011 and included subjects of the D.E.S.I.R. cohort within a longitudinal study aimed at understanding the complex pathophysiology of the metabolic syndrome (138, 139). 16S rRNA gene quantities were measured in 3,280 subjects at baseline and after nine years. Subjects who developed T2D during the duration of the study had significantly higher amounts of bacterial DNA at baseline (odds ratio=1.35, p=0.002). Moreover, bacterial DNA was significantly elevated in subjects with abdominal obesity (odds ratio=1.18, p=0.01). Additionally, pyrosequencing showed the dominant phylum to be Proteobacteria (139), whose amount was found to be an independent risk factor of cardiovascular disease in a subsequent analysis in 3,936 participants of the same cohort (140).

Similarly, blood bacterial DNA composition reminiscent of that observed for the gut microbiome was found in a Japanese cohort of 100 subjects including 50 with T2D. In addition, the detection rate for bacterial DNA was significantly higher in subjects with T2D compared with healthy subjects (28% vs. 4%, p<0.01) (141). In line with above mentioned studies, patients with obesity and liver fibrosis seem to have elevated concentrations of bacterial DNA compared to patients with obesity alone (142). Most recently, Qiu et al. showed that subjects carrying *Bacteroides* had a reduced risk while patients carrying *Sediminibacterium* an increased risk to develop T2D (143). Lammers et al. analyzed the effect of bacterial DNA of eight probiotic strains including Bifidobacterium and Lactobacillus on peripheral blood mononuclear cells (PBMCs) of healthy donors. All strains increased expression of *IL-1, 6, and 10* at high concentrations of 70 µg/ml with largest changes observed for *B. infantis* showing that bacterial DNA can indeed induce strain specific immune alterations in the host (144).

In a small cohort of 58 subjects with obesity, who had undergone bariatric surgery, Ortiz et al. analyzed the translocation of bacterial DNA before and up to 12 months post-surgery. Bacterial DNA was detected in 32.8% of the patients prior to surgery, but only in 13.8% and 5.2% of patients after 3 and 12 months, respectively (145). More importantly, inflammation, LPS levels and insulin resistance persisted in subjects with a reduced clearance of bacterial DNA in the blood even after significant weight loss following bariatric surgery with multivariate analyses revealing bacterial DNA presence as an independent predictor (145). In summary, there is increasing evidence not only for the presence of bacterial DNA and potentially intact bacteria even in healthy subjects but also for the association between the amount of bacterial DNA and bacterial composition with obesity and related metabolic traits. However, current methods have considerable limitations (see *Limitations* of the review) and further, more elaborative studies are warranted to substantiate initial results.

### Bacteria and Bacterial Products Regulate Adipose Tissue Inflammation Locally

The human adipose tissue is a metabolically active organ with large heterogeneity in cellular composition, function, and expression signatures depending on its anatomic location (146).

#### LPS, Adipocytes, and Adipose Tissue Macrophages

Although there are only a few studies providing evidence for the existence of bacterial components in adipose tissue, their potential impact has been repeatedly demonstrated in cell culture experiments. Toll-like receptors, especially TLR4, which are needed for the recognition of LPS are expressed on adipose tissue macrophages as well as adipocytes (147). Stimulation of adipose tissue macrophages with LPS induces fibrosis *via* TLR4 and the induction of the profibrotic factor Transforming growth factor beta 1 (TGFß1) (148). LPS can also act directly on adipocytes, as demonstrated in numerous studies (149–153). Creely et al. could show that IL-6 and TNF-α are secreted in response to LPS in mature subcutaneous adipocytes from lean individuals and subjects with obesity, whereas inhibition of NF-kB reduced the levels of secreted IL-6 after LPS stimulation (149). These results could be confirmed by Vitseva et al. using subcutaneous abdominal fat from subjects with obesity (150). Further works suggest a role for LPS on modifying glycerol permeability and metabolism of murine 3T3-L1 adipocytes (153) and hint to a role of adiponectin in regulating local inflammation by inhibiting LPS-induced NF-kB activation using primary pig adipocytes (152).

#### Bacterial DNA and Live Bacteria

First evidence that bacteria are indeed able to translocate from the gut to adipose tissue stems from mouse experiments. In a pioneering work, Amar et al. used, among other methods, green fluorescent protein (GFP)-labeled *E. coli* to demonstrate the transmucosal passage in mice on high-fat diet (154). Although some initial studies suggested the presence of bacterial DNA in human adipose tissue as well (155), others could not confirm these results (156). In their work, Zulian et al. isolated DNA from whole adipose tissue and mature adipocytes of 14 subjects with obesity. Bacterial PCR products were only observed in the enzymatically isolated adipocytes, but sequencing revealed that 90% of this bacterial DNA belonged to *C. histolyticum*, from which the collagenase enzyme used for adipocytes isolation is derived. All subsequent experiments including culturing experiments were negative (156). In 2017, Udayappan et al. reported the presence of bacterial DNA, mainly stemming from *Actinobacter* and *Ralstonia*, in mesenteric adipose tissue of twelve patients with obesity. However, as they did not include negative controls, contamination as a source of these observed genera could not be excluded (157). Another noteworthy study published by Nakatsuji et al., investigated the subepidermal adipose tissue of six healthy subjects and suggested that the skin microbiome extends to this specific skin-adjacent adipose tissue depot (158). Furthermore, bacterial DNA belonging to several bacteria was observed in the epicardial adipose tissue in six patients with acute coronary syndrome (*Streptophyta* and *Rickettsiale*) and stable angina (*Pseudomonas* and *Moracellaceae*) as compared to subjects with isolated mitral insufficiency. The authors related coronary heart disease with increased bacterial colonization of epicardial adipose tissue and ensuing inflammasone activation (159). More recently, Anhê et al. reported data on contaminant-controlled presence of bacterial DNA in mesenteric, subcutaneous and visceral adipose as well as liver tissue in 40 patients with obesity. Hereby, highest abundance was observed in visceral adipose tissue and liver samples, and patients with T2D had a decreased bacterial diversity. Diversity was especially high in patients with obesity but without T2D in mesenteric adipose tissue (160). Data recently presented by our group could extend and go beyond the proof of bacterial DNA by showing the presence of living bacteria in adipose tissue using catalyzed reporter deposition (CARD) - fluorescence *in situ* hybridization (FISH) methods. Furthermore 16S rRNA gene content of 75 patients with obesity was quantified and sequenced, showing that both bacterial quantity and taxonomy were associated with markers of inflammation and insulin resistance (137). Moreover, miniscule amounts of bacterial DNA (*E. coli*) were sufficient to induce a proinflammatory response in human subcutaneous adipocytes, as observed by a dose-dependent increase of *IL6* and *TNF* expression. Unexpectedly, expression of neither *TLR4* and *TLR9*, nor the NLR family pyrin domain containing 3 (*NLRP3)* inflammasome increased at any concentration (137). Similar direct evidence for the translocation of *via*ble gut bacteria to adipose tissue, in this case mesenteric adipose tissue, was presented by Ha et al. using human biopsies, gnobiotic mice as well as primary cells (161). Bacterial translocation occurred in both healthy subjects and patients with Crohn`s disease (CD), but taxonomic profiles differed. Using gnobiotic mice treated with *C. innocuum*, the bacterium most frequently isolated in CD, Ha et al. evidenced translocation of these bacteria into adipose tissue. This was accompanied by increases in *adiponectin* and Peroxisome proliferator-activated receptor Gamma (*PPARG)* expression and adipose tissue expansion. These results suggest that, at least in the case of CD, specific bacteria including *C. innocuum* can restructure mesenteric adipose tissue leading to the expansion and fibrosis of creeping fat. This might be a defense mechanism to prevent systemic translocation of bacteria beyond this compartment (161).

#### Impact of Bacterial Metabolites on Adipose Tissue Function

SCFA are recognized by multiple G-protein coupled receptors (GPR), and receptors GPR41, 43 and 81 are known to be expressed in adipose tissue (162). First evidence for an adipose tissue specific effect of SCFA was observed by Kimura et al., who showed that mice with a global GPR43 knockout were obese compared to wild type controls whereas mice with a selective overexpression of GPR43 in adipose tissue were leaner (163). The authors further demonstrated that GPR43 stimulation with acetate improved glucose and lipid metabolism in adipose tissue, but not in muscle and liver (163), thereby confirming prior results by Robertson et al., that showed reduced lipolysis in adipose tissue after stimulation of GPR43 with acetate (164). These results were recently strengthened by experimental treatment of multipotent human adipose tissue-derived stem cells with either mixtures of butyrate, propionate and acetate or the individual SCFA in varying concentration between 1 µmol/l and 1 mmol/l. Whereas mixtures high in butyrate had no effect on glycerol release, mixture with high concentration of acetate and propionate decreased basal glycerol release (165). Treatment with only butyrate even increased glycerol release slightly but significantly, whereas isolated treatment with acetate reduced glycerol release (165). Next to their effects on lipolysis, acetate and propionate also seem to influence adipogenesis *via* GPR43, as shown by Hong et al. in 3T3-L1 cells (166). The authors found no GPR43 expression in the stromal vascular fraction (SVF) or preadipocytes, but expression levels increased gradually with adipogenesis. Furthermore, siRNA mediated silencing of GPR43 blocked adipocyte differentiation (166). A further study analyzed the effect of SCFA-rich HFD compared to normal HFD diet on adipose tissue biology: Supplementation of SCFA increased expression of GPR43 in adipose tissue and reduced its expression in the colon. Next to decreased leptin and increased adiponectin expression in adipose tissue, the authors showed an increase in adipose tissue beigeing under SCFA treatment (167).

### Pancreas and Liver: Bacterial Actions on Central Metabolic Organs

#### LPS Effects on Liver and Pancreas

The liver has been shown to participate in active LPS clearance. Increased amounts of endotoxin are still not without consequence for the liver: As a response to LPS uptake and stimulation, hepatocytes express acute phase protein serum amyloid A, which further promotes LPS clearance (168). However, hepatocytes can also utilize the TLR4 cascade to take up LPS (169, 170). That being said, it is assumed that altered TLR4 signaling is a key factor in metabolic liver diseases including NAFLD (171). TLR4 is expressed on various liver cell types including hepatocytes, monocytes, Kupffer cells and stellate cells (172). Using primary human liver biopsies and human hepatocytes (HepaRG), it was shown that LPS increases NF-kB translocation and activity by ~2.5 fold, although the observed effect was only partially mediated by TLR4 as demonstrated by siRNA knockdown and chemical blocking of TLR4 (173). Kheder et al. stimulated macrophages (J774) and HepG2 hepatocytes with LPS, which led to increased TNF-α expression (**Figure 1E**). Co-treatment with increasing doses of vitamin D3 and docosahexaenoic acid (DHA) reduced these effects in a dose dependent manner (174). Lee et al. used mouse hepatocytes deficient for TLR4, Myeloid differentiation primary response 88 (MyD88) and TIR-domain-containing adapter-inducing interferon-β (TRIF) to show that LPS induces hepcidin expression, an antimicrobial and iron regulating protein, *via* TLR4 in a MyD88 dependent manner (175).

On the other hand, endotoxins have been shown to exert varying effects on various pancreatic cells: Endocrine cells of the human pancreatic islets express CD14 and TLR4. CD14 was synthesized and secreted by SV40-transformed islet cells (HP62) after treatment with LPS. LPS was shown to regulate glucose-dependent insulin secretion and induced an inflammatory response (176). By analyzing isolated human and rodent islets, Amyot et al. showed that LPS impairs insulin expression, whereby human islets are more sensitive to this effect. LPS further decreases the expression of pancreatic and duodenal homeobox 1 (PDX-1) and Transcription factor Maf (MafA) and this inhibition is prevented by blocking NF-kB but not p38 MAPK signaling, linking bacterial induced inflammation with pancreatic dysfunction (177).

#### Bacterial Translocation to the Liver

Bacterial DNA has also been evidenced in liver tissue under contamination control (160). More recently, Sookoian et al. were able to localize LPS derived from Gram-negative bacteria in the portal tract and evidenced a diverse repertoire of bacterial DNA in the liver, the composition of which was driven in part by obesity. This metataxonomic signature was furthermore related to histological findings in NAFLD, with expansion of proteobacterial DNA being associated with lobular and portal inflammation as well as liver cirrhosis in non-morbidly obese subjects. Morbid obesity on the other hand displayed wider associations to several taxa thought to contribute to more detrimental findings (178).

#### SCFA Impact on Liver Function and Pancreas

Most of the SCFA taken up from the colon are metabolized by the liver and only small proportions enter the peripheral circulation (about 20% for butyrate and propionate) (179–181). Treatment of HepG2 cells with SCFA ranging from C3 to C6 increased Apolipoprotein A (ApoA-I) expression in a dose-dependent matter whereas secreted ApoA-I to medium was reduced. Additionally, butyric acid increased carnitine palmitoyltransferase I (CPT1) expression, a key protein of beta-oxidation, and increased activity of PPARA (180). Butyrate was also shown to improve insulin sensitivity and increase energy expenditure in mice, and mice on HFD treated with butyrate exhibited higher protein levels of cyclic 5’ AMP-activated protein kinase (cAMPK), P38 and PGC-1a in the liver compared to HFD mice without treatment (182). Sahuri-Arisoylu et al. analyzed the effect of acetate, showing that acetate decreased lipid accumulation and improved hepatic function. The authors observed decreased levels of circulating aspartate transaminase and alkaline phosphatase, as well as reduced expression of genes involved in lipogenesis, such as fatty acid synthetase (183). Acetate also protects rodent models against diet-induced weight increase by altering liver metabolism, as Kondo et al. showed by increased expression levels of fatty acid oxidation enzymes as well as PPARA in the liver (184).

As in adipose tissue, GPR43 is also expressed on pancreatic β-cells, and expression is increased in mice fed with HFD. GPR43 knockout in mice leads to impaired insulin secretion and treatment of murine and human islet cells with a GPR43 agonist leads to increased intracellular Ca2+ and inositol triphosphate levels as well as increased insulin secretion (185). In contrast, a study concurrently did not evidence any effects on glucose homeostasis in GPR43-/- mice on normal chow or HFD compared to wild type mice. In *ex vivo* studies, presence of acetate potentiated insulin secretion (186). Similarly, it was shown that propionate potentiated dynamic glucose-stimulated insulin secretion *in vitro* and further protected human islets from inflammatory cytokine and sodium palmitate induced apoptosis (179).

### Impact of Bacteria and Bacterial Products on Skeletal Muscle Metabolism

Evidence on the role of endotoxin on muscle tissue insulin sensitivity mainly stems from septic models. During hyperinsulinemic euglycemic clamps, 4 ng LPS per kg body weight were administered to healthy donors, resulting in an increased peripheral glucose uptake as well as increased circulating concentrations of norepinephrine and cytokines (180). Concurrently Reyna et al. evidenced an elevated TLR4 expression in muscle tissue of subjects with obesity and T2D and concentrations of TLR4 protein isolated from muscle tissue correlated positively with insulin resistance (181). Furthermore, patients exhibited lower IκBα content and increased expression of *IL6* and *SOD2* and these results could be replicated in primary human myotubes by treatment with palmitate (181). More functional approaches were, among other performed by Liang et al., who similarly differentiated primary human muscle cells into myotubes and treated them with LPS. Endotoxin induced inflammation and reduced insulin signaling; effects which were counteracted by a treatment with the TLR4-Inhibitor (182). These results are supported by data published by Frisard et al. on treatment of mice and human myotubes with high and low concentrations of LPS, thereby reflecting septic or metabolic endotoxemia conditions and showing an increased glucose utilization and reduced fatty acid oxidation in skeletal muscle (183). Low levels of endotoxin are sufficient to modulate mitochondrial consumption and substrate oxidation preferences, an effect which was absent in the presence of specific antioxidants, thereby suggesting a role of reactive oxygen species in mediating observed effects (184).

Houghton et al. tested the effect of various microbiome catabolites, including phenolic compounds, bacterial metabolites, and their phenolic conjugates. Several of these compounds increased glucose uptake to differentiated human skeletal myoblasts (LHCN-M2) as well as overall metabolism rates (187). Most prominent effects were observed for isovanillic acid 3-O-sulfate (IVAS) and dihydroferulic acid 4-O-sulfate. IVAS furthermore promoted upregulation of GLUT1, GLUT4, and phosphoinositid-3-kinase (Pi3K) p85α proteins (187).

The effect of SCFA on skeletal muscle metabolism and function was recently reviewed in an excellent publication by Frampton et al. (188). Studies examining direct effect on skeletal muscle cells are mainly using rat-derived L6 myotubes or mouse-derived C2C12 myotubes and no human studies were found. Briefly, treatment with SCFA increased fatty acid oxidation (about 30% for 0.5 mM butyrate (189)) and fatty acid uptake (i.e., using 0.5 mM acetate) in L6 myotubes (190). Furthermore, stimulation with acetate and propionate promotes enhanced insulin-independent uptake of glucose to both mouse and rat myotubes (190, 191). Maruta et al. also report an upregulation of GLUT4 gene expression as well as protein levels upon treatment with 0.5 mM acetate (190).

## Limitations

It can be assumed that subjects not suffering from sepsis have lower bacterial load and display lower endotoxemia making careful handling of samples to avoid contamination, careful data interpretation to avoid over-reporting of false positive results as well as method standardization allowing comparability and reproducibility, paramount aspects for planning studies contributing to the subject as well as interpretation of the available literature. Here, we highlight some concerns, which should be considered in interpretation of relevant literature.

### Measurement of Intestinal Permeability

There are various methods to measure the integrity of the intestinal barrier with differing degrees of sensitivity and specificity: Methods include Ussing chambers (87), histology and electron microscopy for biopsies and transepithelial/transendothelial electrical resistance (TEER) for cell culture experiments (192). The gold standard for *in vivo* studies is the lactulose/mannitol (La/Ma) or similar dual sugar tests, which assess the flow of indigestible sugars from gastrointestinal tract to the circulation and the combination of different sugars allows a location specific measurement (34, 193). In addition, biomarkers such as calprotectin, alpha-1-trypsin, fatty acid binding protein, and zonulin are used. There is extensive literature on benefits and disadvantages of each method (34, 193–196). However, in our opinion, studies employing biomarkers are generally lacking in strength due to various reasons: a) correlations with dynamic permeability tests such as dual sugar tests or Ussing chambers are not present or very weak; b) most of these markers are also acute phase proteins and are therefore increased in inflammation; c) their actions are not limited to the gut paracellular pathway; d) commercially available ELISAs are often not validated, as we and others recently demonstrated that the preferred ELISA for the most commonly used biomarker zonulin measures different products (197, 198). Consequently, relevant literature should be interpreted carefully in this regard (199).

### Endotoxin

Measurement of endotoxin, in particular at low concentrations, is very challenging and prone to errors, leading to considerable discussion pertaining to the significance of the test and its interpretation. One major aspect to be considered is that LPS are not actively excreted by bacteria but that release of LPS only takes place after gram negative bacterial cell death and lysis, which in turn does not justify the wide use of LPS as a surrogate marker for “live” bacterial translocation. However, there could still be an intrinsic value for the increased exposition of the host to bacteria in general. Measurement of LPS has routinely been done using the Limulus Amoebocyte Lysate (LAL) assay. Although the FDA has recognized the validity of approaches using the LAL method, LAL reagents are not required to be obtained from an FDA-licensed manufacturer when used in the context of research, making standardization between methods reported in the literature almost impossible. Beyond this, conducting the LAL assay is highly challenging. This is related to the magnitude of chemical and physical products and factors, which are known to interfere with the test’s ability to detect LPS. These factors include pre-analytical conditions such as sampling, where plastic or siliconized ware can lead to endotoxin absorption (200) and render LAL assay ineffective. Moreover LPS quantification presumes sampling in pyrogen/LPS free ware, which can be done *via* dry heat sterilization at high temperature, a widely ignored factor possibly contributing to the immense range of LPS from 0.01 to 60 EU/ml reported in healthy subjects in the literature (120, 201). Furthermore, various factors within the blood can interfere with LPS testing, including bile salts, lipoproteins, EDTA and heparin (202), which makes pretreatments of samples to measure LPS unavoidable. Moreover, using different units impedes the interpretation of findings from different studies (203): whereas some studies report LPS levels in weight per water volume (pg/mL), others report endotoxin units per unit volume (EU/mL), which reflects LPS activity. Additionally, when considering that host-derived proteins binding of LPS largely modulates its activity and clearance from the circulation (204–206), it becomes evident that whatever is measured after pretreatment of the samples to overcome “low LPS recovery” might not reflect *in vivo* conditions at all. This is best exemplified by the fact that there is poor concordance between endotoxemia and gram-negative bacteremia and that endotoxemia is detected in less than 50% of subjects with gram-negative sepsis (207). Independent from units, most studies reported a 0.5 to 2-fold increase of LPS in subjects with obesity compared to subjects with normal weight, as clarified by Boutagy et al. (203). Consequently, they propose using fold changes (in disease vs. controls) instead of absolute values to describe the contribution of endotoxemia to the study setting until unified and comparable methods are established.

In addition, LPS derived from different bacteria can show large heterogeneity in the O-antigen polysaccharide with consequence on the inflammatory potential and biological function of endotoxin (133, 134). This point is normally neglected in studies on endotoxin and should therefore be considered more often in new studies.

### Bacterial DNA

In studies analyzing bacterial DNA in samples with low bacterial biomass, be it quantification or sequencing, contamination is a major problem, which is well-highlighted by the recent debate about a possible placenta microbiome (208–211). Contamination can arise from multiple sources (air, skin, reagents) and during all experimental steps. Furthermore, even sterile lab ware can contain traces of bacterial DNA, as per definition a sterile environment is only defined by the absence of living microorganisms (212). The use of adequate negative controls and the deployment of subsequent bioinformatic tools to address them become eminent to avoid reporting false positive results (213). Although these problems are increasingly addressed by the research community, a set of consistent and widely accepted approaches would help for the general comparability and reproducibility of data.

### Data Interpretation

In most works on metabolic endotoxemia it is assumed that LPS is the consequence of impaired intestinal barrier. However, other locations can contribute to a metabolic endotoxemia as well. For instance, it was shown that oral interventions like extraction of teeth or periodontal probing but also everyday actions such as chewing and oral hygiene can lead to an influx of endotoxin and bacteria into the circulation (214, 215). It was also observed that subjects with obesity are more prone to suffer from gingivitis, which is most likely due to increased insulin resistance (216, 217). It is therefore likely that LPS and bacteria from the oral cavity contribute more to systemic ‘bacterial burden’ and possibly inflammation than has been previously assumed. Additionally, the skin microbiome extends to various compartments, thus implicating a potential impact for this compartment in translocation to subsequent tissues (158). However further studies are desirable to support these findings. Furthermore, since LPS is rapidly cleared from the circulation in the liver, which is also the first organ reached by LPS taken up from the gastrointestinal tract, whatever measureable endotoxin under various conditions points toward a chronic influx and the additional contribution of other origins like oral cavity, lungs, or the skin (**Figure 1F**).

## Summary/Conclusion

In this review, we focused on causes and possible consequences of impaired intestinal permeability, thereby focusing on obesity and components of metabolic diseases. Consequences include translocation of endotoxin, bacterial DNA, or live bacteria as well as bacterial metabolites to the circulation, a process often associated with the term metabolic endotoxemia or bacteremia. Starting with animal studies, there now is also compelling evidence for an impairment of the human intestinal barrier in diseases such as T2D and obesity. Consequently, increased circulating bacterial load is now a well-established hallmark of metabolic diseases and published data suggest that impairment of the gut barrier can trigger and further aggravate metabolic impairment. A less studied subject is the presence and effect of bacteria in other host tissues, including liver, muscle, pancreas, and adipose tissue.

However, as shown in this review, several studies suggest that tissue bacteria or components and metabolites thereof either reflect or directly contribute to the development and progression of metabolic diseases. Underlying mechanisms such as TLR4 dependent activation of NF-kB have been introduced but a holistic approach encompassing the complexity of host immune factors is widely lacking. There has also been compelling underreporting of shortcomings in the methods used such as LPS measurement, making claims toward causality rather elusive. Furthermore, only a few studies were published on quantification and sequencing of bacterial DNA in host tissues, which have been similarly plagued with false positive results partly due to contamination.

In conclusion, it seems unavoidable that our bacterial inhabitants also contribute to the modulation of their metabolic environment shaping the body’s responses to nutrients and contributing ultimately to disease as has been shown for the gut microbiota in recent years (218). Considering the current relevance of microbiome research in understanding health and nutrition and the promising avenues for therapy and prevention, it will be inevitable to revisit many of the notions introduced here to allow for robust and reliable mechanistic approaches.

## Author Contributions

LM, PK, and RC designed the review. LM and RC interpreted literature and drafted the manuscript. LM designed the figure. MB and PK critically reviewed and edited the manuscript. All the authors read and approved the final manuscript. All authors contributed to the article and approved the submitted version.

## Funding

LM was funded, and this work was supported by a grant from the Deutsche Forschungsgemeinschaft (DFG, German Research Foundation—Projektnummer 209933838—SFB 1052; B03). The authors acknowledge support from the German Research Foundation (DFG) and Universität Leipzig within the program of Open Access Publishing.

## Conflict of Interest

The authors declare that the research was conducted in the absence of any commercial or financial relationships that could be construed as a potential conflict of interest.

## Glossary

**Table:**

TLR	toll-like receptor
T2D	type 2 diabetes
TJ	tight junctions
IgA	immunoglobulin A
PKC	protein kinase C
MAPK	mitogen-activated protein kinase
INF-γ	interferon gamma
TNF-α	tumor necrosis factor alpha
PI-3K/Akt	Phosphoinositide 3-kinases/Protein kinase B pathway
NfkB	nuclear factor kappa-light-chain-enhancer of activated B cells
ERK	extracellular signal-regulated kinase
ARF6	ADP-ribolysation factor-6
HIF-1α	Hypoxia-inducible factor 1-alpha
GLUT1; 2; 4	glucose transporter
Cxcr	CXC-Motiv-Chemokine receptor
HFD	high fat diet
db/db	leptin receptor deficient mouse model
ob/ob	leptin gene overexpression mouse model
ZO	zonula occludens
PAI-1	Plasminogen-Aktivator-Inhibitor 1
STAMP2	six transmembrane protein of prostate 2
IL-1, 6, 10	interleukin 1, 6, 10
La/Ma	lactulose mannitol
HOMA-IR	homeostatic model assessment for insulin resistance
NAFLD	Non-alcoholic fatty liver disease
NASH	non-alcoholic steatohepatitis
MLN	mesenteric lymph nodes
SCFA	short chain fatty acids
LPS	lipopolysaccharide
sCD14	soluble cluster of differentiation 14
LDL	low-density lipoprotein
HDL	high-density lipoprotein
LDL-R	LDL receptor
iFABP	as intestinal fatty acid binding protein
LBP	LPS binding protein
DJB	duodenojejunal bypass surgery
FITC	Fluorescein isothiocyanate
TGF-β1	Transforming growth factor beta 1
GFP	green fluorescent protein
CARD-FISH	catalyzed reporter deposition fluorescence *in situ* hybridization
NLRP3	NLR family pyrin domain containing 3
PPARG	Peroxisome proliferator-activated receptor gamma
CD	Crohn’s disease
GPR	G protein-coupled receptor
SVF	stroma vascular fraction
DHA	docosahexaenoic acid
MyD88	Myeloid differentiation primary response 88
TRIF	TIR-domain-containing adapter-inducing interferon-β
PDX-1	pancreatic and duodenal homeobox 1
MafA	Transcription factor Maf
cAMPK	cyclic 5’ AMP-activated protein kinase
C3	complement component C3
ApoA1	Apolipoprotein A1
SOD2	Superoxide dismutase 2
mitochondrial, IVAS	isovanillic acid 3-O-sulfate
ELISA	enzyme-linked immunosorbent assay
LAL	Limulus Amoebocyte Lysate
EU/ml	endotoxin units per ml
FDA	United States Food and Drug Administration

*Terms are ordered as they appear in the text for the first time.
